# Patient with Undifferentiated Schizophrenia and Gender Incongruence, a Case Report

**DOI:** 10.1192/j.eurpsy.2025.2303

**Published:** 2025-08-26

**Authors:** V. Gaberšek, N. Rijavec, I. Rahne Otorepec

**Affiliations:** 1 University Psychiatric Clinic Ljubljana, Ljubljana, Slovenia; 2 Outpatient Clinic for Sexual Health, University Psychiatric Clinic Ljubljana, Ljubljana, Slovenia

## Abstract

**Introduction:**

We represent a case report of a female patient with schizophrenia and gender identity disorder, shedding light on the complexity of her medical condition. The patient gave the informed consent for anonymous presentation of her case.

**Objectives:**

We want to highlight the unique vulnerability of schizophrenia patients with comorbid gender identity issues. The diagnostic and treatment processes of these patients should be insightful, careful and interdisciplinary.

**Methods:**

Descriptive report of a case report based on the regular examination of the patient, review of patients clinical file and a non-systematic literate review.

**Results:**

The 23-year-old female patient, diagnosed with undifferentiated schizophrenia at the age of 18, has been receiving regular one-monthly therapy with aripiprazole long-acting injection since 2019. The psychosis has been in stable remission since 2019. Due to comorbid mixed anxiety and depressive disorder, she was receiving sertraline. In 2021, the patient identified as a transgender male. He was admitted to outpatient clinic for sexual health, where he was diagnosed with gender identity disorder. In February 2023, the patient started receiving testosterone transdermal gel due to the recommendation letter of the Slovenian interdisciplinary team for gender identity confirmation. Three months later, he reported insomnia and strong intrapsychic tension; intense hands tremor was also observed. He was voluntarily admitted to the psychiatric clinic due to suspected psychotic symptoms.

**Conclusions:**

Before prescribing hormone therapy to the patient in our case, the psychotic background of his desire for gender reassignement was excluded by multidisciplinary team. Due to suspected relaps of schizophrenia, which appeared three months after the patient had started receiving testosterone treatment, the Slovenian interdisciplinary team for gender identity confirmation reconsidered the patient’s case and decided to temporarly discontinue his testosterone hormone therapy. The patient continues with his psychiatric treatment.

It is important to notice that exacerbation of psychotic symptoms in the course of schizophrenia may prevent implementation of various stages of treatment of gender dysphoria. Hormonal pharmacotherapy can affect the patients emotional state. The use of pharmacological and surgical methods used for gender reassignment should be precluded in the case of current psychotic process. Treatment of gender dysphoria should be conducted in accordance with current version of the World Professional Association of Transgender Health standards.

**Disclosure of Interest:**

None Declared

